# miR-196a Ameliorates Cytotoxicity and Cellular Phenotype in Transgenic Huntington’s Disease Monkey Neural Cells

**DOI:** 10.1371/journal.pone.0162788

**Published:** 2016-09-15

**Authors:** Tanut Kunkanjanawan, Richard L. Carter, Melinda S. Prucha, Jinjing Yang, Rangsun Parnpai, Anthony W. S. Chan

**Affiliations:** 1 Yerkes National Primate Research Center, 954 Gatewood Rd. N.E., Atlanta, GA, 39329, United States of America; 2 Department of Human Genetics, Emory University School of Medicine, 615 Michael St., Atlanta, GA 30322, United States of America; 3 Embryo Technology and Stem Cell Research Center, School of Biotechnology, Suranaree University of Technology, Nakhon Ratchasima 30000, Thailand; University of Florida, UNITED STATES

## Abstract

Huntington’s disease (HD) is an inherited neurodegenerative disorder caused by the expansion of polyglutamine (polyQ) tract that leads to motor, cognitive and psychiatric impairment. Currently there is no cure for HD. A transgenic HD nonhuman primate (HD-NHP) model was developed with progressive development of clinical and pathological features similar to human HD, which suggested the potential preclinical application of the HD-NHP model. Elevated expression of miR-196a was observed in both HD-NHP and human HD brains. Cytotoxicity and apoptosis were ameliorated by the overexpression of miR-196a in HD-NHP neural progenitor cells (HD-NPCs) and differentiated neural cells (HD-NCs). The expression of apoptosis related gene was also down regulated. Mitochondrial morphology and activity were improved as indicated by mitotracker staining and the upregulation of *CBP* and *PGC-1α* in HD-NPCs overexpressing miR-196a. Here we demonstrated the amelioration of HD cellular phenotypes in HD-NPCs and HD-NCs overexpressing miR-196a. Our results also suggested the regulatory role of miR-196a in HD pathogenesis that may hold the key for understanding molecular regulation in HD and developing novel therapeutics.

## Introduction

HD is an autosomal dominant neurodegenerative disorder caused by the expansion of CAG trinucleotide repeats located at the first exon of the *HTT* gene [1–4]. Clinical features of HD include cognitive, psychological, and motor deficits [5–9]. Molecular instability, a core component in disease pathogenesis and progression, has been investigated by transcriptomic and small RNA profiling approaches [10–14]. Dysregulation of genes and non-coding RNA such as micro RNAs (miRNAs) in the brain are highly correlated with neuropathological changes in diseases such as HD [13,15–22]. Dysregulated expression of peroxisome proliferator-activated receptor γ (PPARγ) co-activator 1α (*PGC-1α*), a regulator of mitochondrial biogenesis and oxidative stress [23,24]; CREB binding protein (*CBP*), a histone acetyltransferase (HAT) transcriptional co-activator [25,26] and brain-derived neurotrophic factor (*BDNF*) are all important for the maintenance and survival of neurons [27,28] and are all dysregulated in HD. In addition to transcriptomic dysregulation, alteration of miRNA expression level has also been reported in neurological disorders including psychiatric disorders, autism spectrum disorder, Alzheimer’s disease, Parkinson’s disease and HD [16,29–31]. A recent miRNA profiling study on human HD prefrontal cortex identified five miRNAs that are located in the *Hox* gene cluster and were upregulated in HD when compared to control [17]. Three of the five miRNAs (miR-196a-5p, miR-196b-5p and miR-615-3p) have near zero levels in the control which suggested their potential as a biomarker for HD [17]. Among these three candidates, overexpression of miR-196a ameliorates spinal and bulbar muscular atrophy (SBMA) [32] and HD [15] cellular and clinical phenotypes that suggested the therapeutic potential of miR-196a. Moreover, miR-196a was also highly expressed during early cancer development and is a potential early cancer biomarker [19,33]. Although the *HTT* gene is not a putative target of miR-196a, miR-196a targets genes that are involved in neuronal differentiation, neurite outgrowth [34], cell death and survival that further suggested its role in HD pathogenesis [32,34–36]. Among these mRNA targets, *Anexin-1A* (*ANXA1*) is a mediator of apoptosis and inhibitor of cell proliferation [37,38]. The relation between miR-196a and *Hox* gene cluster further suggested its involvement in neuroprotective response in HD [17,21].

This study evolved based on the recent development of the HD-NHP model [39,40] and the development of HD-NCs from iPSCs derived HD-NPCs [40–42]. HD-NHPs develop progressive decline in cognitive, behavioral and motor functions as they age [43–46]. Transcriptional dysregulation of mRNA and miRNA in peripheral blood and brain tissues was also observed in HD-NHPs [14,30]. HD-NHP brains revealed the formation of mutant HTT (mHTT) aggregates and nuclear inclusion which further suggested the potential of HD-NHP as a large animal model for studying HD pathogenesis [40,41]. Additionally, HD-NPCs and their derivative neural cells develop HD cellular phenotypes including the accumulation of mHTT aggregates, nuclear inclusion, mitochondrial dysfunction and increase susceptibility to oxidative stress [41]. Most importantly, HD cellular phenotypes in HD-NPCs and neural cells can be reversed by genetic and biochemical approaches, which suggested the potential of HD-NPCs as an *in vitro* platform for studying HD pathogenesis and drug discovery research [41]. Here we use HD-NPCs and HD-NCs to investigate if the over expression of miR-196s can ameliorate or rescue HD cellular phenotypes including cell viability, apoptosis, mitochondrial functions and dysregulated gene expression.

## Materials and Methods

No live animal or human subjects were used in the study. All brian tissues were acquired through non-profit brain bank or resource sponsored by NIH. No IACUC or IRB are needed for the use of the biomatierals.

### Monkey and human brain tissues

Brain tissues of HD1, HD7 and WT monkey were provided by the brain bank of the Transgenic Huntington’s Disease Monkey Resource (THDMR) sponsored by the ORIP at NIH. Three groups (Control x 4, HD/Stage 1 x 4 and HD/Stage 3 x 4) of human striatal samples with four individuals for each group were acquired from the Emory Alzheimer’s disease Research Center and the Emory Neuroscience NINDS Core Facilities (ENNCF). Individuals were between 52 to 67 years of age with an average age for the control, HD/Stage 1 and HD/Stage at 61, 60.75 and 62.5 years of age, respectively. HD stages were determined based on clinical assessment.

### Establishment of HD-NPCs over expressing miR-196a

A lentiviral vector carried miR-196a under the control of Tet-On inducible system (Tet-hsa-miR-196a) with zeocin resistant gene regulated by human polyubiquitin promoter (Ubi-zeo) placed downstream of the Tet-hsa-miR-196a (pLV-miR-196a) was used in this study. High titer LV-miR-196a was prepared by the co-transfection of 0.68 ug of pVSVG, 1.014 ug of Δ8.9, and 1.35 ug pLV-miR-196a into 293FT cell [7,39]. Two days after transfection, supernatant was collected, concentrated by ultracentrifugation and followed by infection of wild-type NPCs (WT-NPCs) and HD-NPCs. NPCs were seeded at 20,000 cells/cm^2^ the day before infection. On the day of viral infection, NPCs culture was replaced with fresh neural proliferation medium (NPM; Neurobasal-A medium (Life Technologies) supplemented with B27 (Life Technologies), penicillin/streptomycin (P/S), 2 mM L-glutamine, basic fibroblast growth factor (bFGF) (R&D, 20 ng/ml), and mouse leukemia inhibitory growth factor (mLIF) (Chemicon, 10 ng/ml)) with concentrated virus supplemented with 8 ug/mL polybrene (Sigma) for two days followed by replacement with NPM supplemented with 100 ug/mL zeocin for selection. Expression of miR-196a was determined by quantitative RT-PCR (qPCR) using has-miR-196a TaqMan probe (Applied Biosystems).

### Neural progenitor cell culture and neural differentiation

WT-NPCs and HD-NPCs were derived from NHP pluripotent stem cell [41]. Maintenance and neural differentiation of NPCs was performed as described by Carter and colleagues [41]. In brief, NPCs were cultured on polyornithine/laminin (P/L) coated tissue culture dish with NPM medium. NPCs were dissociated by using Accutase^®^ (Life Technologies).

To *in vitro* differentiate NPCs to neural cells, 30,000 NPCs/cm^2^ were seeded onto P/L coated tissue culture dish. Neural proliferation medium was replaced with neural differentiation medium (NDM; DMEM/F12 (Life Technologies) supplemented with P/S (Invitrogen), 2 mM L-glutamine, 1x N2 (Invitrogen), 1x B27 (Life Technologies), and 0.1 mM 2-Mercaptoethanol (Sigma)) for four days. On day 5, 0.2 μg/mL Sonic Hedgehog (SHH; R&D) and 0.1μg/mL Fibroblast growth factor-8 (FGF-8; R&D) were supplemented into NDM for four days. On day 8, 200 mM ascorbic acid (Sigma) was then added into the medium until the end of neural differentiation on day 21 [41].

### Quantitative real-time PCR (qPCR)

Total RNA from tissue and cell samples was prepared by using TRIzol® (Life Technologies). Genomic DNA was removed by using Turbo DNA-free Kit (Life Technologies) according to the manufacturer’s instructions. Total RNA (200 μg) was reverse transcribed using an RNA-to-cDNA kit (Applied Biosystems). HD related genes (*HTT exon 1*, *HTT exon 26*, and *Hip-*1), apoptosis gene (*ANXA1*), mitochondrion related genes (*CBP* and *PCG1α*) and *BDNF* were analyzed by using qPCR technique with SsoAdvaned Universal SYBR Green Supermix (Bio-Rad).

For miR-196a, 300 ug of total RNA was transcribed using TaqmanMiroRNA Reverse Transcription Kit (Applied Biosystems) with hsa-miR-196a and RNU6B RT primer. qPCR was performed using TaqMan® gene microRNA expression primers. In this experiment CFX96 Real-Time Detection System (Bio-Rad) was used. qPCR primer sequences are listed in S1 Table.

### Immunocytochemistry

NPCs and neural cells cultured on P/L-coated glass slide were fixed with 4% paraformaldehyde (PFA) for 15 minutes followed by two times washed with PBS. Fixed cells were permeabilized by incubation with blocking solution (0.2% Triton-X-100 (Sigma) and 3% BSA in PBS) for 1 hr. Samples were then incubated with primary antibodies overnight at 4°C followed by three washes in PBS and incubation with secondary antibody for 1 hr at room temperature. Images were captured by using CellSens software (Olympus). To quantify the ratio of positive cells, 10 images were taken randomly and counted by using CellSens software (Olympus). Primary and secondary antibodies are listed in S2 Table.

### Cell stress and apoptosis assays

For cell viability, NPCs were seeded in P/L coated 96 wells plate the day before treatment. Cell viability was determined by using MTT assay (ATCC) according to manufacturer’s instructions.

Cell cytotoxicity was measured by using Vybrant Cytotoxicity Assay Kit (Life Technologies) according to the manufacturer’s instructions to measure the release of the cytosolic enzyme glucose-6-phosphate dehydrogenase (G6PD) from damage cells into culture media.

For cleaved caspase-3 protein expression, cells were fixed and immunostained with cleaved caspase-3 specific antibody (Millipore).

### Mitochondria morphology

Mitochondria with active mitochondrial membrane potential were determined by using Mitotracker® Green FM (Life Technologies). Cells were then fixed and nuclei were labeled by using Hoechst 33342. Images were acquired using a Zeiss LSM 510 NLO META confocal microscope (Oberkochen, Germany).

### Statistical analysis

All experiments were comprised of three biological replicas. Statistical analysis was performed by using SPSS 12.0 (SPSS, Inc., USA). Data was represented as mean ± standard error. Statistical differences were calculated using two tailed unpaired T-tests and Analysis of variance (ANOVA). Difference were illustrated as * = p < 0.05, ** = p < 0.01, and *** = p < 0.001.

## Results

### Upregulation of miR-196a in HD-NHP brain, NPCs and NCs

**Fig 1:**
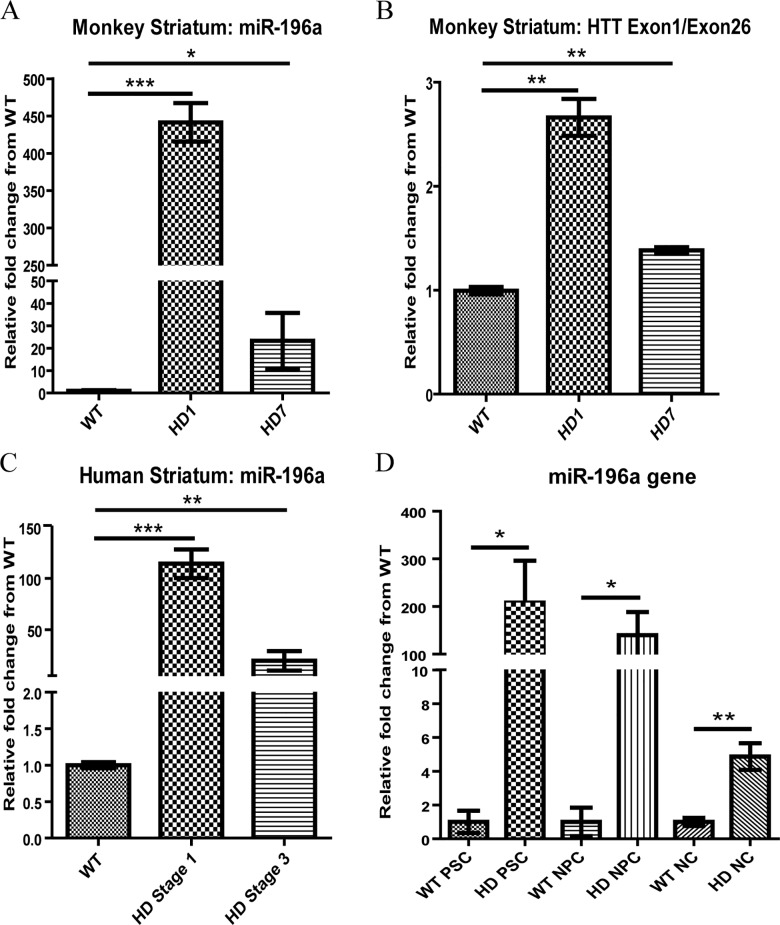
Expression of miR-196a and *mHTT* in HD monkeys and humans brain. (A) Expression level of miR-196a in each single WT, HD1, and HD7 monkey striatum was illustrated by relative fold change to WT monkey striatum. (B) *mHTT* transgene expression in HD1 and HD7 monkey striatum was illustrated by relative fold change to the expression level of endogenous exon 26 and compared to HTT level of WT monkey striatum. Quantitative gene expression assay of monkey brain tissues was determined by three technical replicate measurements. (C) Expression of miR-196a in human control (n = 4), HD stage 1 (n = 4) and stage 3 (n = 4) patient striatum were illustrated by relative fold change to control brain tissues. (D) Expression of miR-196a in WT and HD monkey cells (PSCs, NPCs and NCs) was illustrated by relative fold change to corresponding WT cells. The result was generated from a single cell line derived from the same WT and HD monkeys. Three independent samples of different passages were collected at different times and used for RNA preparation for gene expression analysis. miR-196a was measured by Taqman microRNA assays (ABI). Results are presented as mean±SEM. Data was analyzed by using one-way ANOVA (A, B and D) and 2 tailed unpaired T test for 1C (* p < 0.05, ** p < 0.01, and *** p < 0.001).

Expression of miR-196a and *mHTT* transgene in HD1 and HD7-NHP striatum were measured by qPCR (Fig 1A and 1B) and both exhibited higher expression levels when compared to the WT-NHP (Fig 1A and 1B). Measurement of *mHTT* transcript was determined by the relative expression levels of the exon 1 of *HTT* gene (both *mHTT* and endogenous *HTT*) to exon 26 (endogenous *HTT*). These findings were consistent with miR-196a expression pattern in human HD striatum where miR-196a was significantly increased in Stage 1 and 3 (n = 4) HD patients when compared to the controls (n = 4) (Fig 1C). Upregulation of miR-196a was also observed in undifferentiated pluripotent stem cells (PSCs), NPCs, and NCs derived from HD-NHPs when compared to the controls (Fig 1D).

### Impact of overexpressing miR-196a in NPCs and NCs

**Fig 2:**
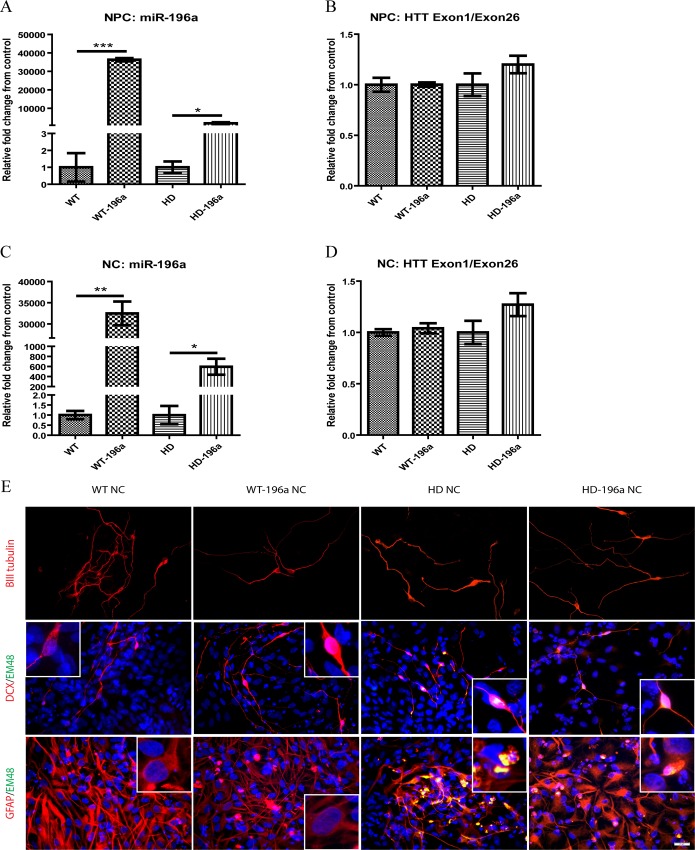
Overexpressing miR-196a in monkey NPCs and NCs. (A) Expression of miR-196a in NPCs overexpressing miR-196a was illustrated by relative fold change to parent WT or HD NPCs. (B) Mutant *HTT* gene expression in NPCs overexpressing miR-196a was determined by using *HTT* exon 1 specific primer set. Expression level was illustrated by relative fold change to parent WT or HD NPCs. (C) Expression of miR-196a in *in vitro* differentiated neural cells (NCs) overexpressing miR-196a was illustrated by relative fold change to parent WT-NCs or HD-NCs. (D) Mutant *HTT* gene expression in *in vitro* differentiated NCs overexpressing miR-196a was determined by using *HTT* exon 1 specific primer set. Expression level was illustrated by relative fold change to parent WT-NCs or HD-NCs. The qPCR assays were performed with three biological replicas for each gene of interest. (E) Immunotaining of WT, WT-196a, HD and HD-196a NCs. Antibodies specifically recognized neuronal markers (βIII tubulin, Doublecortin (DCX) and GFAP), and mHTT (mEM48) were used. The images showed here were representative images captured from three independent studies. Data was analyzed by two tailed unpaired T test. Results are presented as mean±SEM (* p < 0.05, ** p < 0.01, and *** p < 0.001).

Overexpression of miR-196a in WT-NPCs and HD-NPCs was confirmed by quantitative measurement in WT-NPCs and HD-NPCs with (WT-196a NPCs and HD-196a NPCs) or without (WT-NPCs and HD-NPCs) introduced with miR-196a transgene by lentiviruses. miR-196a expression was significantly increased in NPCs of WT-196a NPCs and HD-196a NPCs when compared to WT-NPCs and HD-NPCs, respectively (Fig 2A).

Elevated expression levels of miR-196a in WT-NPCs and HD-NPCs did not affect the NPC property based on the immunostaining of NPC markers including Sox-2, Pax-6, Musashi-1 and Nestin (S1A Fig) and NC differentiation (Fig 2E). Additionally, the expression of *mHTT* was not affected by the overexpression of miR-196a as expected, since *HTT* is not a putative target of miR-196a (Fig 2B). To further determine the relationship between *HTT* and miR-196a, we analyzed miR-196a expression in *HTT* knocked down HD-NPCs (shHD) that was previously described [41] and compared it with WT-NPCs and HD-NPCs. There was no difference in the expression of miR-196a in shHD and HD-NPCs and were both significantly higher than WT-NPCs (S1B Fig). Our next step was to determine if overexpressing miR-196a affect neural differentiation. All NPCs with or without overexpressing miR-196a were *in vitro* differentiated into NCs [41]. We confirmed the overexpression of miR-196a in NCs derived from both WT-196a NPCs and HD-196a NPCs when compared to those derived from WT-NPCs and HD-NPCs (Fig 2C). However, the overexpression of miR-196a has no effect on the expression of *mHTT* (Fig 2D). Immunostaining using neural specific antibodies demonstrated the expression of neuronal markers in all groups (Fig 2E). Our results further suggested the overexpression of miR-196a did not affect NPC property and NC differentiation.

### Effect of miR-196a on gene expression in HD-NPCs and HD-NCs

**Fig 3:**
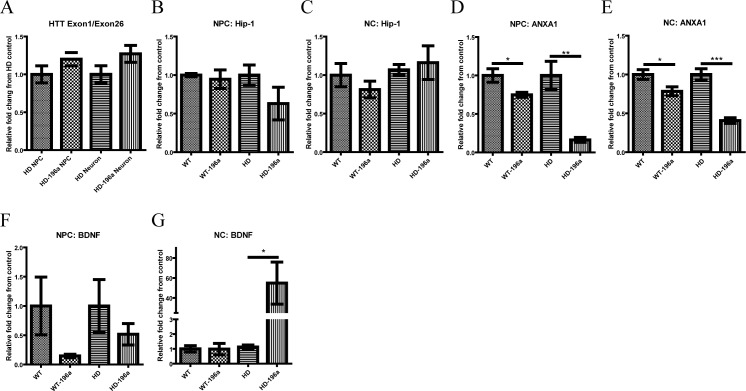
Dyregulated genes in HD-NPCs and HD-NCs overexpressing miR-196a. (A) Expression of *mHTT* in HD and HD-196a NPCs and NCs. (B-C) Expression of *Hip-1* in WT, WT-196a, HD and HD-196a NPCs (B) and NCs (C). (D-E) Expression of *ANXA1* in WT, WT-196a, HD and HD-196a NPCs (F) and NCs (G). (F-G) Expression of *BDNF* in WT, WT-196a, HD and HD-196a NPCs (F) and NCs (G). The expression level was illustrated by relative fold change on the expression level of miR-196a expressing cells to parent WT-NPCs or HD-NPCs, and WT-NCs or HD-NCs, respectively. qPCR assays were performed in three biological replicate measurements of a single NPC, or NC isolate from a single animal. Data was analyzed by two tailed unpaired T test. Results are presented as mean±SEM (* p < 0.05, ** p < 0.01, and *** p < 0.001).

To determine the impact and the role of miR-196a in HD pathogenesis, a panel of genes related to HD (*HTT*, Huntingtin-interacting protein 1:*HIP-1*), apoptosis (Annexin1A: *ANX1A*) and neural cell growth (*BDNF*) were quantitatively measured by qPCR in NPCs and NCs. Comparative gene expression between WT-NPCs and HD-NPCs and differentiated NCs were shown in S2A–S2N Fig). The expression of *mHTT* was not different in HD-NPCs (Fig 2B) and HD neurons (Fig 2D) with or without overexpressing miR-196a (Fig 3A). *HIP-1* is a pro-apoptotic protein [47] that interacts with HTT, and its expression was also not affected by the overexpression of miR-196a in both NPCs and NCs (Fig 3B and 3C). *ANX1A* is a mediator of apoptosis and inhibitor of cell proliferation which has been reported in various cancer types [48,49] and is a putative target of miR-196a [37]. Significant reduction in *ANX1A* was found in WT- and HD-NPCs and NCs overexpressing miR-196a (Fig 3D and 3E) that suggested the neuroprotective effect of miR-196a. Finally, *BDNF* is one of the neural growth factors that is highly dysregulated in HD and resulted in synaptic dysfunction in the brain [27]. The expression level of *BDNF* was not different in WT- and HD-NPCs (S2G Fig) but *BDNF* expression in HD-NCs was significantly lower compared to WT-NCs (S2H Fig). Although the expression of *BDNF* was not significantly changed in HD-196a NPCs, it was significantly elevated in HD-196a NCs (Fig 3G and 3H).

### Ameliorate cytotoxicity and apoptosis in NPCs and NCs by miR-196a

**Fig 4:**
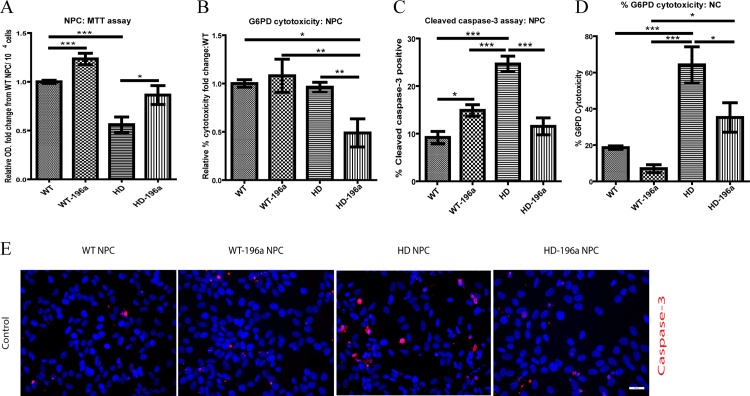
miR-196a ameliorated cytotoxicity and apoptosis in NPCs and NCs. (A) Viability of WT, WT-196a, HD and HD -196a NPCs was determined by MTT assay. (B) Cytotoxicity of WT and HD NPCs with or without overexpressing miR-196a was determined by G6PD assay. The G6PD cytotoxicity was calculated by percentage changes from WT-NPCs. The MTT and G6PD assay were performed in three biological replicas with three technical replicas. (C) WT, WT-196a, HD and HD-196a NPCs were immunostained with cleaved caspase-3 antibody. Percentage of the number of cleaved caspase -3 positive NPCs was calculated based on five random images from two biological replicas with at least 1200 total cell counts. (D) Cytotoxicity of NCs was determined by G6PD assay. WT-NCs *vs* HD-NCs with or without overexpressing miR-196a. (E) Immunostaining of WT, WT-196a, HD and HD-196a NPCs using cleaved caspase-3 specific antibody. Data was analyzed by one-way ANOVA. Results are presented as mean±SEM (* p < 0.05, ** p < 0.01, and *** p < 0.001).

To investigate if the overexpression of miR-196a can ameliorate cytotoxicity and apoptosis in HD-NPCs and HD-NCs, MTT assay and G6PD cytotoxicity analysis were performed. Overexpression of miR-196a in both WT-NPCs and HD-NPCs significantly improve viability at similar levels that suggested improvement in NADH production as shown by MTT assay (Fig 4A). The cytotoxicity reduction effect of miR-196a was determined by G6PD cytotoxicity assay. No significant effect was observed on cytotoxicity in WT-NPCs with or without the overexpression of miR-196a (Fig 4B). However, the overexpression of miR-196a in HD-NPCs showed a beneficial effect on cell cytotoxicity compared with HD-NPCs without overexpressing miR-196a (Fig 4B). To further confirm if miR-196a improves apoptosis in NPCs, WT- and HD-NPCs were immunostained with antibody that specifically recognized cleaved caspase-3 followed by cell count analysis. (Fig 4C and 4E). In WT-NPCs, overexpression of miR-196a increased apoptosis (Fig 4C) while a significant reduction in cleaved caspase-3 positive cells was observed in HD-NPCs overexpressing miR-196a (Fig 4E). Since the neuroprotective effect of miR-196a was clearly shown in HD-NPCs, our next step was to determine if a similar effect was found in neural cells. Cytotoxicity in HD-NCs was significantly higher compared to WT-NCs and the overexpression of miR-196a ameliorated cytotoxicity in both WT-NCs and HD-NCs (Fig 4D). Overall, we demonstrated the overexpression of miR-196a could ameliorate cytotoxicity and apoptosis in HD-NPCs and HD-NCs.

### Effect of miR-196a on mitochondrial morphology and functions

**Fig 5:**
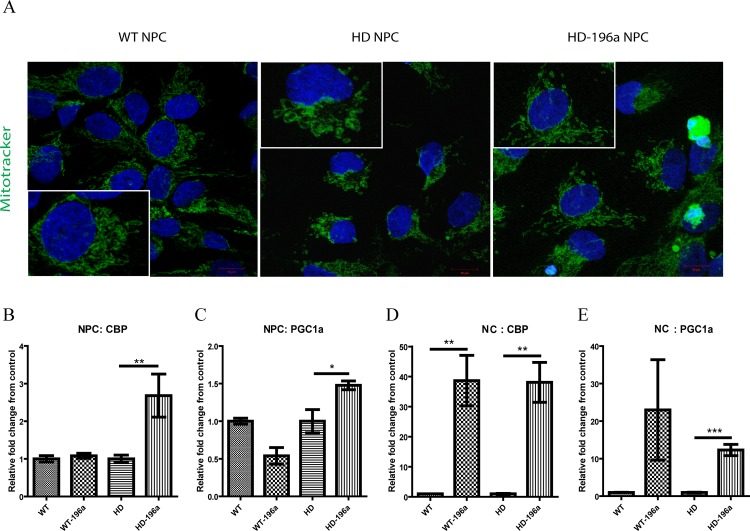
miR-196a improved mitochondrial morphology and gene expression. (A) WT, HD and HD-196a NPCs was stained with Mitotracker® Green FM (green) and nuclei were labeled using Hoechst 33342 (blue). The image shown here is representative of images captured from three images of independent experiments. (B-E) quantitative measurement of *CBP* and *PCG1α* transcripts in WT, WT-196a, HD and HD-196a (B, C) NPCs and (D, E) NCs. The expression level of *CBP* (B and D) and *PGC1α* (C and E) was illustrated by relative fold change on the expression level of miR-196a overexpressing cells to parent WT-NPCs or HD-NPCs, and WT-NCs or HD-NCs, respectively. The qPCR assays were performed in three biological replicas. Data was analyzed by two tailed unpaired T test. Results are presented as mean±SEM (* p < 0.05, ** p < 0.01, and *** p < 0.001).

Mitochondrial dysfunction has been demonstrated in HD animal models and patients that include the alteration of calcium buffering capacity [50], impaired bioenergetics [51,52], increased oxidative stress and effects on fission and fusion homeostasis [53]. A recent report by Song and colleagues [54] demonstrated mitochondrial fragmentation in HD neurons and fibroblasts. Impaired mitochondrion were fragmented with shorter and round-shape morphology which was resulted from the increased mitochondrial fission rates over mitochondrial fusion in HD neurons [54]. NPC mitochondrial morphology was examined by staining with Mitotracker (Molecular Probe) and imaged by using confocal microscopy. In HD-NPCs, short and fragmented mitochondrion surrounding the nucleus (Fig 5A, middle panel) suggested mitochondrial fission, which was morphologically different from the elongated thread of mitochondrion in WT-NPCs which suggested a balance ratio of mitochondrial fission and fusion (Fig 5A, left panel). When miR-196a was overexpressed in HD-NPCs, an increase in elongated thread of mitochondrion was observed which suggested the increase of mitochondrial fusion (Fig 5A, right panel). To further confirm if mitochondrial function was improved by miR-196a, the expression levels of genes that are related to mitochondrial functions and are dysregulated in HD were quantitatively measured. Down regulation of *PGC-1α*, a regulator of mitochondrial biogenesis and oxidative stress [23,24] and CREB binding protein (*CBP*), a histone acetyltransferase (HAT) transcriptional co-activator that affect *PGC-1α* [25,26,55] have been reported in HD. Upregulation of *CBP* and *PGC-1α* was observed in HD-NPCs (Fig 5B and 5C) and HD-NCs (Fig 5D and 5E) overexpressing miR-196a. Similar expression pattern for *CBP* and *PGC-1α* was also observed in WT-NCs except that *PGC-1α* was not significantly increased in WT-NCs expressing miR-196a (Fig 5E). Our results suggested that miR-196a might improve mitochondrial function by the upregulation of *CBP* and *PGC-1α* to promote oxidation phosphorylation and reduce oxidative stress, which was consistent with the amelioration of cytotoxicity by miR-196a.

## Discussion

HD is a neurodegenerative disorder caused by expanded polyglutamine tract at the N-terminus of the HTT protein. The expansion of polyQ tract in HTT protein elicits systemic impact on the central nervous system and peripheral tissues [56,57] which include dysregulation in global gene and miRNA expression [14,19], proteome [58], apoptosis [47,59] and mitochondrial biogenesis [52,54]. Based on miRNA array study on WT and HD monkey brains, miR-196a was one of the dysregulated miRNAs that was also found to be dysregulated in human HD brains [17]. Clinical benefits of miR-196a have been reported in neurodegenerative disorder such as SBMA via targeted silencing of CUGBP, Elav-like family member 2 (CELF2) [32], and human immunodeficiency virus type 1(HIV-1) associated neurodegeneration by interfered HIV-1 trans-activator of transcription (Tat) protein [35]. Amelioration of HD phenotypes was also observed in HD mouse and stem cell models [15,17]. Recently, bioinformatics analysis showed potential mechanism of miR-196a significantly altered ABC transporter, RIG-I like receptor pathway, immune system, tissue remodeling and cytoskeleton remodeling in HD rodent model [34]. However, the role of miR-196a in HD pathogenesis has not yet been fully understood while HTT is not the direct target of miR-196a. We have shown that HD-NPCs and HD-NCs exhibited transcriptomic dysregulation, increase apoptosis and cytotoxicity similar to HD cellular phenotypes [29,40,41,60]. Most importantly, these HD specific cellular phenotypes can be reversed by genetic and chemical treatment which suggested the potential of this progenitor cell model for studying the pathogenic role of miR-196a in HD [41]. Here we used the HD-NPC model to investigate the impact of miR-196a on HD cellular phenotypes which include susceptibility to oxidative stress, gene dysregulation and mitochondrial abnormality.

Tet-on inducible system was initially designed to control miR-196a expression. Although the induction efficiency reached our expectation, we observed adverse responses include apoptosis and cytotoxicity in NPCs treated with doxycycline. Additionally, the basal expression of the Tet-On construct without doxycycline induction was sufficient to express miR-196a (Fig 2A and 2C) and improve properties of HD-NPCs and HD-NCs as shown in this study. Therefore, we decided not to supplement doxycycline in all studies. The overexpression of miR-196a did not affect progenitor cell properties and neural differentiation capability in both WT- and HD-NPCs (S1 Fig). As expected, the expression of *mHTT* and *Hip-1* was not altered by the overexpression of miR-196a since *HTT* is not the putative target of miR-196a based on targetscan database (http://www.targetscan.org) (Fig 3A–3C). One may argue that the *mHTT* transgene that was expressed in HD-NPCs only carries the exon1 of the *HTT* gene driven by human polyubiquitin C promoter [39,41,46]. Although *mHTT* transgene might not be the target of miR-196a, the expression of endogenous *HTT* gene was also unaltered which suggested both endogenous *HTT* and *mHTT* genes were not affected by the overexpression of miR-196a (Fig 2B).

Transcriptomic dysregulation in HD brain, fibroblast and peripheral blood cells have been reported [14,30,61–63]. Among these dysregulated genes, we selected genes (*ANXA1* and *BDNF*) that were dysregulated in the brain of HD with distinct cellular functions. *ANX1A* is targeted by miR-196a and suppresses apoptosis in cancer cells overexpressing miR-196a [37]. In HD patient blood, *ANXA1* was upregulated in prodromal and symptomatic patients [64]. We also observed the consistent upregulation of *ANXA1* in HD-NPCs and HD-NCs (S2E and S2F Fig). The overexpression of miR-196a in HD-NPCs and HD-NCs can downregulate the expression of *ANX1A* (Fig 3D and 3E) to promote cell survival and reduced apoptosis (Fig 4). Finally, BDNF is one the most important growth factors that promote neural cell growth and survival and has shown to be dysregulated in cell and animal models as well as in HD patients [27,28,65,66]. Expression level of *BDNF* was not significant different in WT and HD-NPCs with miR-196a (Fig 3F) or without miR-196a (S2G Fig) overexpression suggested that miR-196a has no significant impact on BDNF functions in NPCs since *BDNF* is not highly expressed in NPCs compared to NCs. On the contrary, overexpression of miR-196a in HD-NCs enhanced *BDNF* expression, which benefits neural cell survival (Fig 4D)[34]. Improvement in viability was demonstrated by MTT assay and reduced cytotoxicity by G6PD assay in miR-196s expressing NPCs and NCs further suggested the beneficial effect of miR-196a in ameliorating HD cellular defects (Fig 4) [34]. MTT assay is a commonly used method to examine cell viability and proliferation based on the production of NADH and NADPH [67]. A positive result in MTT assay suggested miR-196a over expression enhanced cell survival and proliferation. Moreover, elevation of MTT result also suggested improvement in mitochondrial functions, which was consistent with our findings in the upregulation of *CBP* and *PGC1α* in HD-NPCs and HD-NCs (Fig 5B–5E) and improvement in mitochondrial morphology (Fig 5A). On the other hand, G6PD cytotoxicity assay was used for quantitative measurement of G6PD enzyme activity that was released from dead cells or cells with damaged plasma membrane [68]. Reduction of G6PD cytotoxicity was observed in miR-196a expressing HD-NPCs and HD-NCs (Fig 4B and 4D).

Caspases, cysteine protease characterized by their high specificity for substrates with an aspartic acid at the site of cleavage in the P1 position, play a prominent role in apoptosis [69]. Additionally, caspase-3 inhibition by small molecule demonstrated neuroprotective effect in transgenic HD rat and mouse model [70,71]. Overexpression of miR-196a reduced cleaved caspase-3 production in HD-196a NPCs suggested the anti-apoptotic effect of miR-196a [34] (Fig 4C). It is interesting that a discrepancy was observed in G6PD and the cleaved caspase-3 assay in NPCs. We speculate that different outcomes from the three assays may be due to the nature of samples that were measured. MTT assay examined proliferation and the level of NADH production. G6PD cytotoxicity assay detects the release of the cytosolic enzyme G6PD from cells into the media. Dead cells or cells with compromised membrane integrity undergoing apoptosis will increase the release of G6PD into culture media. Since G6PD assay measures the levels of G6PD release into the media, it is an accumulation of G6PD during culture regardless of the presence of live cells, apoptotic cells and dead cells. Although NPCs are less sensitive to HD toxicity compared to NCs, both HD-NPCs and HD-NCs exhibited reduced cytotoxicity when overexpressing miR-196a. Unlike G6PD assay, apoptosis was determined by cleaved caspase-3 staining which exclusively examined attached apoptotic cells. Interestingly, WT-NPCs seem to respond differently to the overexpression of miR-196a that resulted in cytotoxic effect instead of reduced apoptosis in HD-NPCs. The difference in results may be due to the different between WT, NPC and NC in response to changes in miR-196a expression levels. Overexpression of miR-196a in HD cells may elicit compensatory mechanism that ameliorates HD pathology while overexpression in WT cells may induce apoptosis similar to tumor formation defense mechanism since miR-196a has an anti-tumorigenic function.

Mitochondrial dysregulation in HD has been reported in various HD models [50,52,53,72,73]. Mitochondrial fragmentation in HD was due to the disturbance of mitochondria fusion and fission homeostasis [73]. mHTT has a stronger binding affinity with dynamin-related protein 1 (Drp-1) than WT HTT which resulted in the increase of mitochondrial fission rate in HD patient lymphoblast [51]. Moreover, loss of Drp-1 function by shRNA silencing reduced mitochondrial fragmentation and neuronal cell death [54]. In HD-NPCs overexpressing miR-196a, mitotracker staining of mitochondria demonstrated the reduction of fragmentation in HD-NPCs. Additionally, the expression of *CBP* and *PGC1α*, genes related to mitochondrial biogenesis function were significantly reduced in HD [26,55]. *PGC1α* is a transcriptional coactivator involved in energy homeostasis, adaptive thermogenesis, α-oxidation of fatty acid and glucose metabolism [74]. The expression of *PGC1α* is transcriptional controlled by CBP, which is also depleted in HD [26,34]. The overexpression of miR-196a resulted in an upregulation of both *CBP* and *PGC1α* genes in HD-NPC and HD-NCs (Fig 5B–5E). This result suggested the role of miR-196a in mitochondrial functions. In-depth investigation in mitochondrial homeostasis and biogenesis using HD-NPCs and HD-NCs may lead to future insights in HD pathogenesis and the regulatory role of miR-196a.

Our findings demonstrated the upregulation of miR-196a could be a compensatory response in HD to defend against cell cytotoxicity, apoptosis, transcriptional dysregulation, proteosome and mitochondrial dysfunctions that lead to neuronal cell death. Although dysregulation of *ANXA1*, a putative target of miR-196a, in HD has strongly suggested the role of miR-196a in regulating neural cell response to stress and pathogenic changes in cells such as the accumulation of mHTT aggregates, continued effort in identifying gene targets of miR-196a that were dynamically changed during HD progression will lead to insight on the role of miR-196a in HD pathogenesis. Here, we showed that HD-NPCs and HD-NCs from HD-NHP developed cellular changes and responses similar to those observed in other cell and animal models as well as in HD patient’s brains. Thus HD-NPCs and HD-NCs could be potentially used as an *in vitro* platform for studying HD pathogenesis, drug discovery research, the development of biomarkers and novel therapeutics.

## Supporting Information

S1 Fig Immunocytochemistry assay of WT and HD NPCs with or without overexpressing miR-196a and shHD. (A) Immunostaining of WT, WT-196a, HD and HD-196a NPCs using NPC markers (Sox2, Pax6, Musashi-1, and Nestin). (B) Expression level of miR-196a in WT, HD, and shHD NPCs by Taqman miRNA assay (ABI). The expression level was presented as relative fold change to HD NPCs. The qPCR assays were performed in biological replicas. Data was analyzed by one-way ANOVA 2. Data are represented as mean±SEM (* p < 0.05, ** p < 0.01, and *** p < 0.001).
(TIF)

S2 Fig Comparison of HD related genes expression in WT and HD NPCs and NCs. Expression levels of (A and B) mutant *HTT* gene, (C and D) *Hip-1*, (E and F) *ANXA1*, (G and H) *BDNF*, (I and J) *Caspase-3*, (K and L) *PGC1a* and (M and N) *CBP*. The expression level in HD cells was illustrated by relative fold change to the WT cells, respectively. The expression level was presented as relative fold change to HD NPCs. The qPCR assays were performed in three biological replicas. Data was analyzed by two tailed unpaired T test. Data are represented as mean±SEM (* p < 0.05, ** p < 0.01, and *** p < 0.001).
(TIF)

S1 Table qPCR primer sequences. Primer sequences for quantitative measurement of miRNA and gene expression in NPC and derivative neural cells.
(DOCX)

S2 Table List of antibodies for immunocytochemistry. Antibodies used for immunostaining of NPC and derivative neural cells.
(DOCX)
