# Supplementary figures and images for: miR-196a Ameliorates Cytotoxicity and Cellular Phenotype in Transgenic Huntington’s Disease Monkey Neural Cells
Source: PLoS One. 2016 Sep 15;11(9):e0162788. doi: 10.1371/journal.pone.0162788 (PMC5025087; doi:10.1371/journal.pone.0162788)

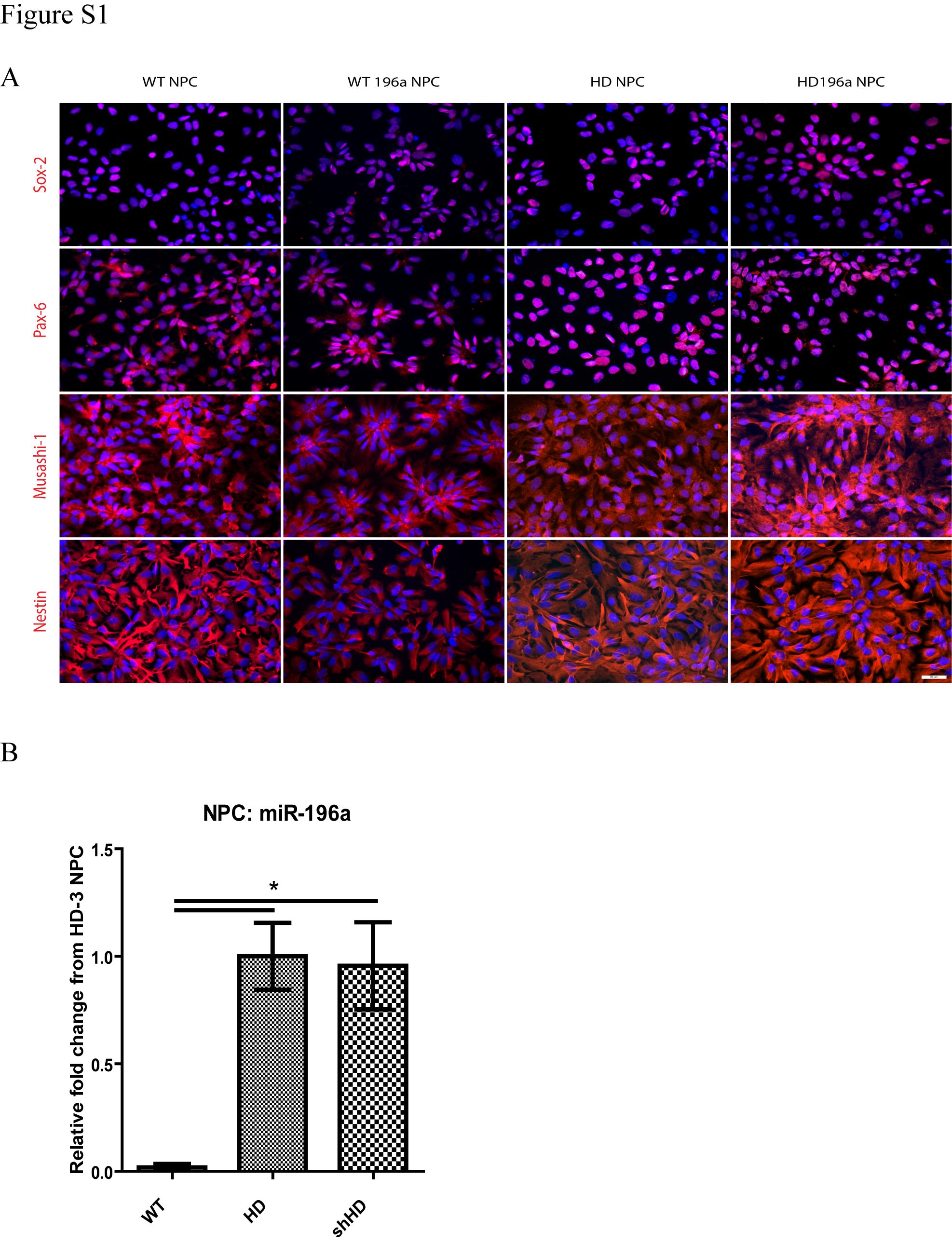

Supplement: S1 Fig — (A) Immunostaining of WT, WT-196a, HD and HD-196a NPCs using NPC markers (Sox2, Pax6, Musashi-1, and Nestin). (B) Expression level of miR-196a in WT, HD, and shHD NPCs by Taqman miRNA assay (ABI). The expression level was presented as relative fold change to HD NPCs. The qPCR assays were performed in biological replicas. Data was analyzed by one-way ANOVA 2. Data are represented as mean±SEM (* p < 0.05, ** p < 0.01, and *** p < 0.001). (TIF) [file pone.0162788.s001.tif]

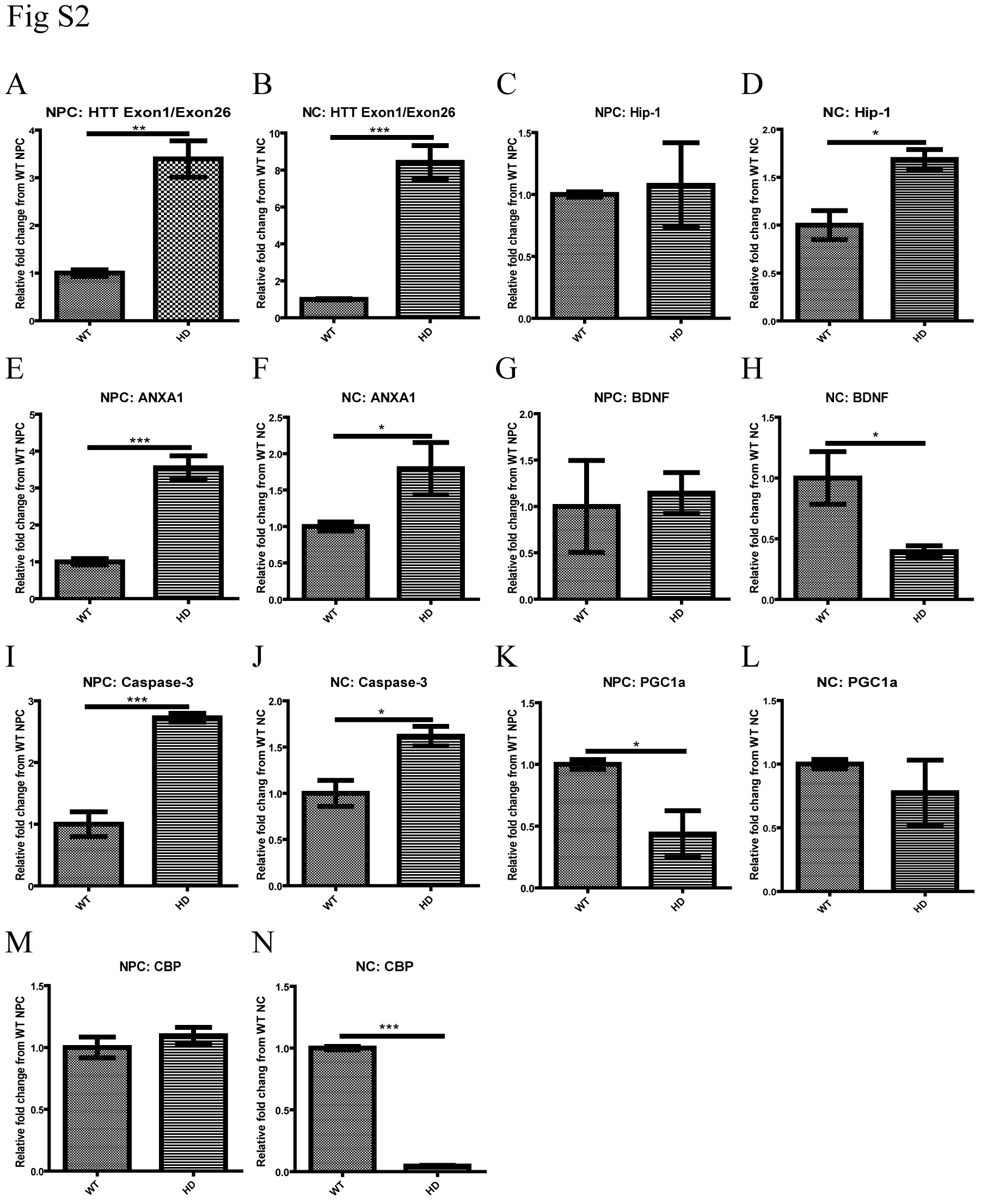

Supplement: S2 Fig — Expression levels of (A and B) mutant HTT gene, (C and D) Hip-1, (E and F) ANXA1, (G and H) BDNF, (I and J) Caspase-3, (K and L) PGC1a and (M and N) CBP. The expression level in HD cells was illustrated by relative fold change to the WT cells, respectively. The expression level was presented as relative fold change to HD NPCs. The qPCR assays were performed in three biological replicas. Data was analyzed by two tailed unpaired T test. Data are represented as mean±SEM (* p < 0.05, ** p < 0.01, and *** p < 0.001). (TIF) [file pone.0162788.s002.tif]
