# Supplementary material for: miR-196a Ameliorates Cytotoxicity and Cellular Phenotype in Transgenic Huntington’s Disease Monkey Neural Cells
Source: PLoS One. 2016 Sep 15;11(9):e0162788. doi: 10.1371/journal.pone.0162788 (PMC5025087; doi:10.1371/journal.pone.0162788)
Supplement: S1 Table — Primer sequences for quantitative measurement of miRNA and gene expression in NPC and derivative neural cells. (DOCX) [file pone.0162788.s003.docx]

**Supplementary table 1. qPCR primer sequences**

| **Taqman ® Primer** | |
| --- | --- |
| **Gene Symbol** | **Taqman ® Primer Context Sequence** |
| RNU6B | CGCAAGGATGACACGCAAATTCGTGAAGCGTTCCATATTTTT |
| hsa-miR-196a | UAGGUAGUUUCAUGUUGUUGG |

| **SYBR Primers** | | |
| --- | --- | --- |
| **Gene Symbol** | **Forward Primer** | **Reverse Primer** |
| **Ubiquitin C** | CCACTCTGCACTTGGTCCTG | CCAGTTGGGAATGCAACAACTTTA |
| **HTT Exon 1** | GCGACCCTGGAAAAGCTGAT | CTGCTGCTGCTGGAAGGACT |
| **Caspase-3** | TCGCTTTGTGCCATGCTG AAA C | TGTTGCCACCTTTCGGTTAACC |
| **BCL2L1** | CGGGATGGGGTAAACTGG | AGGTGGTCATTCAGGTAAGTGG |
| **ANXA1** | ATGAAAGGTGCTGGAACTCG | TCTCCCTTGGTTTCATCCAG |
| **BDNF** | GCC CAATGAAGAAAACAA TAAGG | AGCAGAAAGAGAAGAGG GGC |
| **CBP** | AGCGAAACCAACAAACCATCC | TGGGGTCTATGGGATTTGGGT |
| **PGC1α** | CTAAAGACCCCAAAGGATGC | GCGGTGTCTGTAGTGGCTTG |
