# Supplementary material for: miR-196a Ameliorates Cytotoxicity and Cellular Phenotype in Transgenic Huntington’s Disease Monkey Neural Cells
Source: PLoS One. 2016 Sep 15;11(9):e0162788. doi: 10.1371/journal.pone.0162788 (PMC5025087; doi:10.1371/journal.pone.0162788)
Supplement: S2 Table — Antibodies used for immunostaining of NPC and derivative neural cells. (DOCX) [file pone.0162788.s004.docx]

**Supplementary table 2. List of antibodies for immunochemistry.**

| **Antibody** | **Concentration** | **Vender** |
| --- | --- | --- |
| Nestin | 1:1000 | Millipore |
| Sox2 | 1:500 | Stem Cell Technology |
| Musashi 1 | 1:500 | Millipore |
| Pax6 | 1:300 | Covance, Atlanta, GA |
| β-III tubulin | 1:300 | Millipore |
| Tyrosine Hydroxylase | 1:100 | Millipore |
| Map-2 | 1:500 | Millipore |
| Double cortin | 1:500 | Millipore |
| mEM48 | 1:50 | Gift from Dr. XJ Li |
| Alexa Flour 488 | 1:1000 | Life Technology |
| Alexa Flour 594 | 1:1000 | Life Technology |
| Cy-5 | 1:750 | Life Technology |
